# Supplementary material for: Discovery and pharmacophoric characterization of chemokine network inhibitors using phage-display, saturation mutagenesis and computational modelling
Source: Nat Commun. 2023 Sep 16;14:5763. doi: 10.1038/s41467-023-41488-z (PMC10505172; doi:10.1038/s41467-023-41488-z)
Supplement: Supplementary file 4 — Source Data [file 41467_2023_41488_MOESM4_ESM.zip › SourceData/scripts/xngs_20230809084627.tar!/xngs/analysis/SV_EVAA7_ngsplot.pdf]

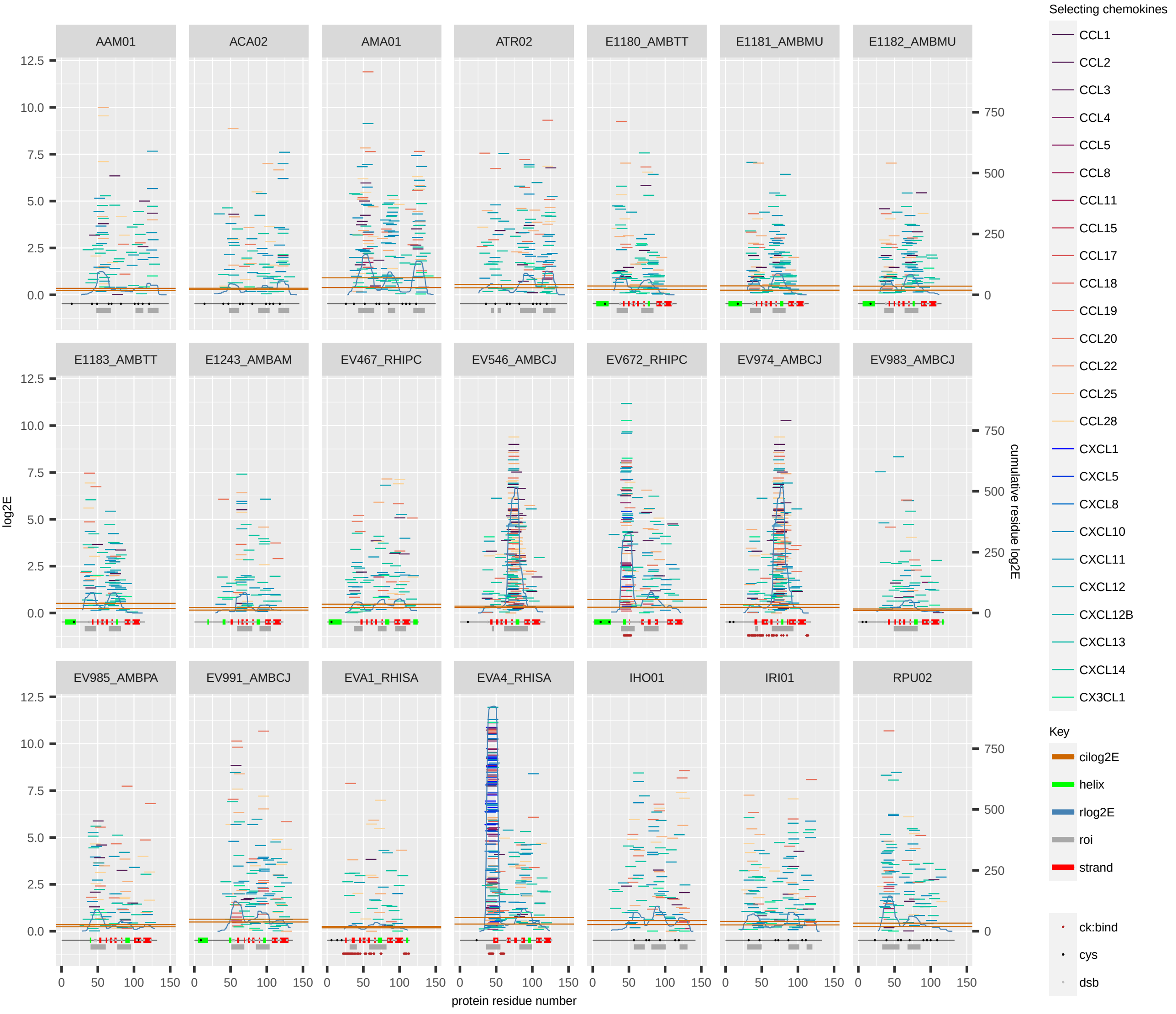

AAM01
ACA02
AMA01
ATR02
E1180_AMBTT
E1181_AMBMU
E1182_AMBMU
E1183_AMBTT
E1243_AMBAM
EV467_RHIPC
EV546_AMBCJ
EV672_RHIPC
EV974_AMBCJ
EV983_AMBCJ
EV985_AMBPA
EV991_AMBCJ
EVA1_RHISA
EVA4_RHISA
IHO01
IRI01
RPU02
log2E
cumulative residue log2E
protein residue number
Selecting chemokines
CCL1
CCL2
CCL3
CCL4
CCL5
CCL8
CCL11
CCL15
CCL17
CCL18
CCL19
CCL20
CCL22
CCL25
CCL28
CXCL1
CXCL5
CXCL8
CXCL10
CXCL11
CXCL12
CXCL12B
CXCL13
CXCL14
CX3CL1
Key
cilog2E
helix
rlog2E
roi
strand
ck:bind
cys
dsb
